# Supplementary material for: Causal Relationship between Aging and Anorexia Nervosa: A White-Matter-Microstructure-Mediated Mendelian Randomization Analysis
Source: Biomedicines. 2024 Aug 16;12(8):1874. doi: 10.3390/biomedicines12081874 (PMC11351342; doi:10.3390/biomedicines12081874)
Supplement: Supplementary file 1 [file biomedicines-12-01874-s001.zip › Supplementary Figure/Figure S4/Singe SNP plot of MR analysis for TL to ubm-b-1801.pdf]

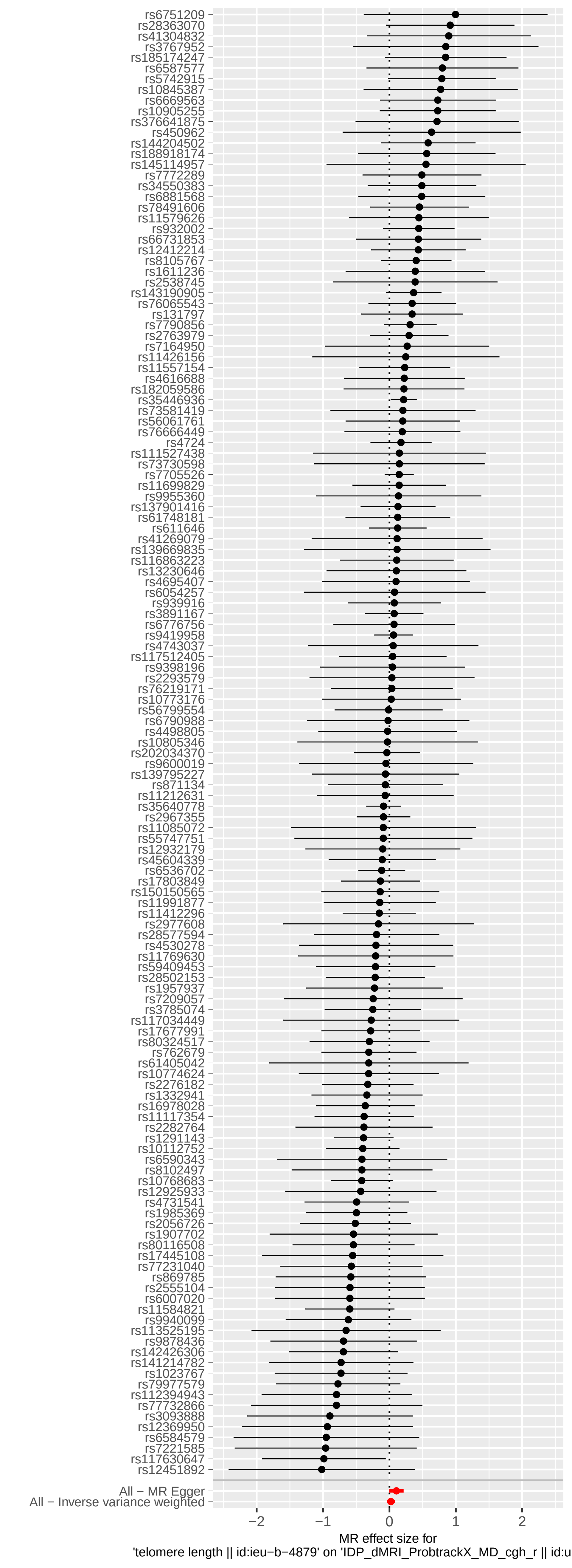

rs6751209
rs28363070
rs41304832
rs3767952
rs185174247
rs6587577
rs5742915
rs10845387
rs6669563
rs10905255
rs376641875
rs450962
rs144204502
rs188918174
rs145114957
rs7772289
rs34550383
rs6881568
rs78491606
rs11579626
rs932002
rs66731853
rs12412214
rs8105767
rs1611236
rs2538745
rs143190905
rs76065543
rs131797
rs7790856
rs2763979
rs7164950
rs11426156
rs11557154
rs4616688
rs182059586
rs35446936
rs73581419
rs56061761
rs76666449
rs4724
rs111527438
rs73730598
rs7705526
rs11699829
rs9955360
rs137901416
rs61748181
rs611646
rs41269079
rs139669835
rs116863223
rs13230646
rs4695407
rs6054257
rs939916
rs3891167
rs6776756
rs9419958
rs4743037
rs117512405
rs9398196
rs2293579
rs76219171
rs10773176
rs56799554
rs6790988
rs4498805
rs10805346
rs202034370
rs9600019
rs139795227
rs871134
rs11212631
rs35640778
rs2967355
rs11085072
rs55747751
rs12932179
rs45604339
rs6536702
rs17803849
rs150150565
rs11991877
rs11412296
rs2977608
rs28577594
rs4530278
rs11769630
rs59409453
rs28502153
rs1957937
rs7209057
rs3785074
rs117034449
rs17677991
rs80324517
rs762679
rs61405042
rs10774624
rs2276182
rs1332941
rs16978028
rs11117354
rs2282764
rs1291143
rs10112752
rs6590343
rs8102497
rs10768683
rs12925933
rs4731541
rs1985369
rs2056726
rs1907702
rs80116508
rs17445108
rs77231040
rs869785
rs2555104
rs6007020
rs11584821
rs9940099
rs113525195
rs9878436
rs142426306
rs141214782
rs1023767
rs79977579
rs112394943
rs77732866
rs3093888
rs12369950
rs6584579
rs7221585
rs117630647
rs12451892
All − MR Egger
All − Inverse variance weighted
−2
−1
0
1
2
MR effect size for
'telomere length || id:ieu−b−4879' on 'IDP_dMRI_ProbtrackX_MD_cgh_r || id:u
